# RAS–Mitogen-Activated Protein Kinase Signal Is Required for Enhanced PD-L1 Expression in Human Lung Cancers

**DOI:** 10.1371/journal.pone.0166626

**Published:** 2016-11-15

**Authors:** Hidetoshi Sumimoto, Atsushi Takano, Koji Teramoto, Yataro Daigo

**Affiliations:** 1 Department of Medical Oncology and Cancer Center, Shiga University of Medical Science, Otsu, Shiga, Japan; 2 Center for Antibody and Vaccine Therapy, Research Hospital, Institute of Medical Science, The University of Tokyo, Tokyo, Japan; H. Lee Moffitt Cancer Center & Research Institute, UNITED STATES

## Abstract

Ectopic programmed cell death ligand 1 (PD-L1) expression in non-small cell lung cancers (NSCLCs) is related to immune evasion by cancer, and it is a molecular target of immune checkpoint therapies. Although some altered signals in NSCLCs are responsible for ectopic PD-L1 expression, the precise mechanisms remain obscure. Because we found a higher frequency of *EGFR/KRAS* mutations in NSCLC cell lines with high PD-L1 expression (p < 0.001), we evaluated the relationships between downstream signals and PD-L1 expression, particularly in three *KRAS*-mutant adenocarcinoma cell lines. The MEK inhibitor U0126 (20 μM) significantly decreased the surface PD-L1 levels by 50–60% compared with dimethyl sulfoxide (p < 0.0001). Phorbol 12-myristate 13-acetate stimulation (100 nM, 15 min) increased (p < 0.05) and two *ERK2* siRNAs as well as *KRAS* siRNAs decreased (p < 0.05) PD-L1 expression. The transcriptional activity of the potential AP-1 site (+4785 to +5056 from the transcription start site) in the *PD-L1* gene was demonstrated by luciferase assays, which was inhibited by U0126. The chromatin immunoprecipitation assay demonstrated the binding of cJUN to the AP-1 site. Two *STAT3* siRNAs decreased PD-L1 expression by 10–32% in two of the three *KRAS*-mutant lung adenocarcinoma cell lines (p < 0.05), while the PI3K inhibitor LY294002 (40 μM) did not change the expression level. Supervised cluster analysis and gene set enrichment analysis between the *PD-L1*-high and -low NSCLCs revealed a correlation between *PD-L1* expression and genes/pathways related to cell motility/adhesion. These results indicate that MAPK signaling is the dominant downstream signal responsible for ectopic PD-L1 expression, in which STAT3 is also involved to some extent. Furthermore, MAPK signaling may control the expression of *PD-L1* and several genes related to enhanced cell motility. Our findings suggest that MAPK, along with STAT3, is important for determining PD-L1 expression, which could be useful for targeted therapies against lung cancers.

## Introduction

Recent advances in immune checkpoint therapies are rapidly changing the clinical applications of cancer therapies [1]. Programmed cell death ligand 1 (PD-L1), also known as cluster of differentiation 274 (CD274) and B7 homolog 1 (B7-H1), is widely expressed in normal tissues (natural killer cells, T and B cells, macrophages, dendritic cells, epithelial cells, and vascular endothelial cells). It is a ligand for programmed cell death 1 (PD-1) receptors expressed on activated T cells [2]. PD-L1/PD-1 interaction is an important immune checkpoint that restricts excessive adaptive immune responses, maintaining immune homeostasis [2]. However, in chronic viral infection or cancers, the continuous exposure of antigen-primed T cells to antigens induces PD-1 on their surfaces. The PD-L1/PD-1 interaction inhibits signals from the T-cell receptor, leading to T cells that are exhausted, a state characterized by being unresponsive to antigens [3].

Ectopic PD-L1 expression has been reported in many different tumor types including lung cancer [4], and it is considered to be one of the mechanisms of immune evasion. Clinical trials have demonstrated the clinical activity of anti-PD-1 or anti-PD-L1 monoclonal antibodies for various tumors including non-small cell lung cancers (NSCLCs), with a response rate of 10% to 30% [5]. Although the significance of PD-L1 expression as a biomarker in cancers is controversial, it should be considered in the context of an immune evasion network created by cancer cells. An immunosuppressive microenvironment is a complex and dynamic state involving various molecules and cells, which originates from constitutively altered signals within cancer cells. These signals also regulate proliferation or metastasis and constitute oncogenic signals as evidenced in *STAT3* [6] or mutated *BRAF* (V600E) [7]. Several driver oncogene products can be molecular targets for cancer immunotherapy and are now under vigorous investigation [8].

The mechanisms of ectopic PD-L1 expression have been examined in various cancers [9–19]. However, the signal pathways responsible for its expression have been found to differ among various cancers. For NSCLCs, *EGFR* mutation [10,11], *EML4-ALK* rearrangement [13], or microRNAs [12] have been reported to regulate PD-L1 expression. The MAPK signal against a background with these mutations has been reported to contribute to PD-L1 expression [13], but the precise mechanism involved in this has not yet been demonstrated. Here we examined the signaling pathways that regulate PD-L1 expression in *KRAS*-mutant NSCLCs and demonstrated that MAPK signaling and downstream AP-1 are crucial for PD-L1 expression.

## Materials and Methods

### Cell lines

NCI-H522, NCI-H1373, NCI-H1650, NCI-H1975, NCI-H23, NCI-H358, NCI-H441, NCI-H647, SK-LU-1, NCI-H226, NCI-H2170, SKMES-1, DMS114, NCI-H460, and BEAS-2B were purchased from American Type Culture Collection. ABC-1, RERF-LC-MS, VMRC-LCD, EBC-1, LK-2, and SBC-5 were purchased from the Japanese Collection of Research Bioresources Cell Bank. All cell lines except NCI-H647, which was studied immediately after purchase, were authenticated by short tandem repeat DNA typing before submission.

### Quantitative reverse transcription polymerase chain reaction (qRT-PCR)

mRNA expression in the cell lines was quantified by real-time PCR using the TaqMan PCR assay for *PD-L1 (CD274)* or SYBR Green Gene Expression Assays (Applied Biosystems) for *KRAS*, *ERK2*, *STAT3*, *RAC2*, *CDA*, *ANXA3*, *MST1R*, *VAV1*, *FBXL13*, *SH3KBP1*, *TRIP6*, *TGFβ1*, *TNFAIP8*, or *RAET1E* and was analyzed by a relative quantitative method (ΔΔCt method) for the target mRNA, which was normalized by control *β-actin* mRNA and BEAS-2B as a reference control cell.

### Mutational analysis of driver oncogenes

Information on the mutational status of driver oncogenes in the cell lines was obtained from the Cancer Cell Line Encyclopedia (CCLE) provided by the Broad Institute and Novartis Institutes for Biomedical Research (http://www.broadinstitute.org/ccle/home), as well as from Catalogue of somatic mutations in cancer (COSMIC) (http://cancer.sanger.ac.uk/cosmic).

### Flow cytometric analysis

Cells were stained with fluorescein isothiocyanate (FITC)-conjugated mAb specific for PD-L1 (MIH1) or the isotype control IgG (MOPC-21) and propidium iodide (PI) (BD Pharmingen). Cell acquisition and analysis were performed with FACSCalibur and CELLQuestPro software (Becton Dickinson). The relative mean fluorescence intensity (MFI) was calculated using the following equation: PD-L1 MFI/isotype control MFI in the PI-negative fraction.

### Inhibitor and RNA interference (RNAi) experiments

For *in vitro* inhibitor assays, U0126 or LY294002 (Cell Signaling Technology; CST) dissolved in dimethyl sulfoxide (DMSO) (Wako) was added to the cells at a final concentration of 20 or 40 μM, respectively, as well as to the same amount of DMSO as a control, and incubated for 24 h. For RNAi experiments, cells plated at 50–60% confluence in a 6-cm dish were transfected with siRNAs specific for *ERK2* mRNA (#1, 5′-GACACAACACCUCAGCAAU-3′ and #2, 5′-CUAACGUUCUGCACCGUGA-3′), *KRAS* mRNA (#1, 5′-CUCAGGACUUAGCAAGAAGUU-3′ and #2, 5′-CAGUUGAGACCUUCUAAUUGG-3′), or *STAT3* mRNA (#1, 5′-GCCUCAAGAUUGACCUAGA-3′ and #4, 5′-AUAGGAAGGUUUAAGGAGA-3′) as well as a control siRNA (siCtrl) (directed against *firefly luciferase* mRNA) (5′-GUGCGCUGCUGGUGCCAAC-3′) (all obtained from Sigma) at 100 nM with Lipofectamine 2000 (Invitrogen). Forty-eight hours after transfection, cells were harvested for flow cytometry, immunoblot, and qRT-PCR assays.

### Immunoblot analysis

Cells were lysed in RIPA buffer containing Halt Protease and Phosphatase Inhibitor Cocktail (Thermo Scientific). Proteins were separated on 10.0% SDS-polyacrylamide gels. The following primary mAbs were used: phospho-p44/42 MAPK (Thr202/Tyr204) (D13.14.4E, rabbit monoclonal; CST), ERK2 (C14, rabbit polyclonal; Santa Cruz), phospho-AKT (Ser473) (D9E, rabbit monoclonal; CST), AKT (C67E7, rabbit monoclonal; CST), phosphor-STAT3 (rabbit polyclonal, #931; CST), STAT3 (124H6, mouse monoclonal; CST), KRAS (234–4.2, mouse monoclonal; Calbiochem), PD-L1 (E1L3N, rabbit monoclonal; CST), β-actin (8H10D10, mouse monoclonal; CST), and GAPDH (14C10, rabbit monoclonal; CST). Horseradish peroxidase-conjugated goat Ab to rabbit IgG (NA931) or donkey Ab to mouse IgG (NA934) (GE Healthcare) was used as the secondary Ab. Immune complexes were detected with ECL Prime Western Blotting Detecting Reagent (GE Healthcare) and ImageQuant LAS 4000 min (GE Healthcare).

### Phorbol 12-myristate 13-acetate (PMA) stimulation analysis

Cells were conditioned with DMSO or U0126 (20μM) for 1hr, 48 h after serum deprivation, followed by stimulation with 100 nM PMA (Sigma). Fifteen minutes later, cells were harvested for immunoblot and qRT-PCR assays.

### Construction of luciferase gene plasmids

The candidate promoter region [−2403 to +153, relative to the transcription start site (TSS), which was determined by DBTSS (http://dbtss.hgc.jp/)] was amplified from genomic DNA purified from NCI-H1373 cells by PCR with PrimeSTAR GXL DNA Polymerase (Takara) and the following primer set: forward primer, 5′-ACCTGAGCTCGCTAGCAACATGACTCACCTGAGGACAA-3′; and reverse primer, 5′-TATCCTCGAGGCTAGCTCCATCCCAAAGAAAGGGTGTAG-3′. The PCR product was subcloned upstream of the luciferase gene of pGL4.17 basic luciferase plasmid (Promega) with the In-Fusion HD Cloning Kit (Takara) (PDL-P).

The region within the first intron of *PD-L1* was assessed for the candidate enhancer element of AP-1 binding sites using publicly available chromatin immunoprecipitation (ChIP)-seq data from the UCSC Genomic Browser (http://genome.ucsc.edu). One candidate region containing an AP-1 binding site (+4785 to +5056 relative to TSS) was amplified by PCR with the following primer set: forward primer, 5′-ttGGATCCAGCACAGAAGAGGTGCTCAA-3′ and reverse primer, 5′-ttGGATCCCTTCAGGTGCCATCCTTCAA-3′ (the underlines at the 5′-ends show the BamHI site for subcloning). The PCR product was cloned to the BamHI site downstream of the luciferase gene of PDL-P (PDL-P+E). The cloned DNA fragments were confirmed by sequencing before the assays.

### Luciferase assay

NCI-H1373 cells were dispensed to Nunclon Delta Surface, a 96-well white-bottomed plate (Thermo Scientific, 136101) the day before transfection. Upon transfection, the cell density reached approximately 50%. One hundred nanograms of firefly luciferase plasmid (pGL4.17 basic, PDL-P, or PDL-P+E) and 100 ng of pGL4.74 [*hRluc*/TK] plasmid (Promega), which encodes *Renilla* luciferase driven by a thymidine kinase promoter, were co-transfected with FuGENE6 (Promega) at a FuGENE (μL) to DNA (μg) ratio of 6. Two hours later, DMSO or U0126 (20 μM) was added. Twenty-four hours after the transfection, the firefly and *Renilla* luciferase activities were determined with the Dual-Glo Luciferase Assay System (Promega) and an infinite M200 (TECAN) multiplate reader. After subtracting the background activity, each firefly luciferase activity was normalized using *Renilla* luciferase activity to determine the transfection efficiency.

### ChIP assay

ChIP was conducted for cJun in NCI-H1373 cells. Immunoprecipitation of the predicted AP-1-responsive element was conducted using the ChIP-IT Express Enzymatic Kit (Active Motif) and cJun mAb (60A8, rabbit monoclonal IgG; CST) or isotype control mAb (DA1E, rabbit monoclonal IgG; CST), followed by qRT-PCR with SYBR Green Gene Expression Assays and the following primer set: forward primer, 5′-TCACATTTCAAGCAGGATGACTAAA-3′ and reverse primer, 5′-TGACTCACAGCCACTCTTCCA-3′. The amount of immunoprecipitated AP-1-responsive element was determined using a standard curve constructed from the serial dilution of input DNA, and fold enrichment was calculated using the cJun/isotype control value.

### Supervised cluster analysis and gene set enrichment analysis (GSEA)

Supervised cluster analysis and GSEA were conducted between the *PD-L1*-high and -low lung cancer cell lines using CCLE Analysis Tools: Differential Expression and Gene Set Enrichment Analysis (GSEA) (http://www.broadinstitute.org/ccle/data/browseAnalyses).

### Statistical analysis

Unpaired Student’s *t* test was used for comparison of the two groups. Data are expressed as mean ± standard deviation (SD). The correlation between MFI and RQ of PD-L1 expression was evaluated by Pearson’s correlation test. Chi-square test was used for comparing the frequency of the two groups, and a p-value of less than 0.05 was regarded as significant.

## Results

### High correlation between *PD-L1* mRNA and surface PD-L1 protein levels in human lung cancer cell lines

**Fig 1:**
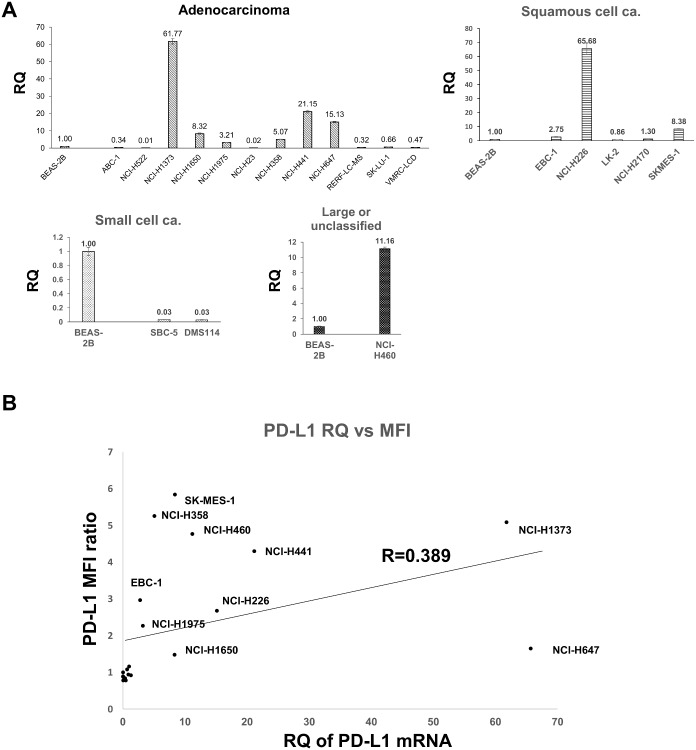
*PD-L1* mRNA levels are correlated to surface PD-L1 protein levels in human lung cancer cell lines. A, Quantitative RT-PCR results of *PD-L1* mRNA in human lung cancer cell lines (histological features: 12 adenocarcinomas, 5 squamous cells, 2 small cells, and 1 large cell) is shown as RQ using the ΔΔCt method with BEAS-2B as a reference. Bars and error bars represent the mean and SD of the technical triplicates, respectively. B, The correlation between the mRNA and surface expression levels of PD-L1. A positive correlation was found between the mRNA and protein levels (Pearson’s correlation coefficient, R = 0.389).

We determined *PD-L1* mRNA levels of a human lung cancer cell line panel as the relative quantity (RQ) to that of BEAS-2B, a human bronchial cell line (Fig 1A) and discriminated between the *PD-L1*-high and -low groups based on the *PD-L1* mRNA level of BEAS-2B. We performed flow cytometry analysis of surface PD-L1 for 20 cell lines from both *PD-L1*-high (*PD-L1* RQ > 2.7) and *PD-L1*-low *(PD-L1* RQ < 1) groups (S1 Fig). MFI of PD-L1 was normalized by that of the isotype control and is shown as the MFI ratio (S1 Table). The correlation between the mRNA and the MFI ratio of PD-L1 was moderate (Pearson’s correlation coefficient, R = 0.389), indicating that transcriptional regulation is important for ectopic PD-L1 expression (Fig 1B). Although our results could not rule out the possible co-existence of a post-transcriptional regulatory mechanism, our observation that all *PD-L1*-high mRNA (R > 2.7) cell lines expressed a substantial amount of PD-L1 on their surfaces suggests that post-transcriptional regulation is not a dominant mechanism regulating PD-L1 levels.

### Higher PD-L1 expression in lung cancer cells is associated with a higher frequency of *EGFR* or *KRAS* mutation

We obtained information about the mutational status of major driver oncogenes or a tumor suppressor gene in the 20 human lung cancer cell lines from the CCLE. The PD-L1-high group contained two *EGFR*-mutant and five *KRAS*-mutant cell lines, the histological characteristics of which resulted six of them being classified as adenocarcinomas and one as a large cell carcinoma, while the PD-L1-low group contained only two *KRAS*-mutant adenocarcinoma cell lines (Table 1). Because the mutations found in other oncogenes (*ALK*, *RET*, *ROS1*, and *ERBB2*) reside outside known functional domains or potential phosphorylation sites, the functional significance of these mutations cannot be specified. The frequency of either *EGFR* or *KRAS* mutations in the PD-L1-high group was significantly higher than that in the PD-L1-low group (p = 0.00056). We obtained the same result about mutational status of *EGFR* or *KRAS* by COSMIC database. We inferred that the downstream signals of *EGFR* or *KRAS* might regulate PD-L1 expression.

**Table 1:** Mutational status of human lung cancer cell lines.

	Cell line	Histology	EGFR	KRAS	ALK	BRAF	MET	RET	ROS1	ERBB2	PTEN
**PD-L1 high**	NCI-H1373	Ad	wt	G12C	p.E1435del^a^	wt	wt	wt	wt	wt	wt
	NCI-H441	Ad	wt	G12V	wt	wt	wt	wt	wt	wt	wt
	NCI-H647	Ad	wt	G13D	wt	wt	wt	wt	wt	wt	wt
	NCI-H1650	Ad	p.ELREA746del	wt	wt	wt	wt	wt	wt	wt	wt
	NCI-H358	Ad	wt	G12C	wt	wt	wt	wt	wt	wt	wt
	NCI-H1975	Ad	L858R, T790M	wt	wt	wt	wt	wt	wt	wt	wt
	NCI-H226	Sq	wt	wt	wt	wt	wt	wt	wt	wt	wt
	SK-MES-1	Sq	wt	wt	wt	wt	wt	wt	wt	wt	wt
	EBC-1	Sq	wt	wt	wt	wt	wt	wt	wt	wt	wt
	NCI-H460	La	wt	Q61H^b^	wt	wt	wt	wt	wt	wt	wt
**PD-L1 low**	SK-LU-1	Ad	wt	G12D	wt	wt	wt	wt	wt	wt	wt
	VMRC-LCD	Ad	wt	wt	wt	wt	wt	wt	wt	wt	wt
	ABC-1	Ad	wt	wt	wt	wt	wt	wt	wt	wt	wt
	RERF-LC-MS	Ad	wt	wt	wt	wt	wt	wt	wt	wt	wt
	NCI-H23	Ad	wt	G12C	wt	wt	wt	R77L^c^	wt	wt	wt
	NCI-H522	Ad	wt	wt	wt	wt	wt	wt	wt	wt	wt
	NCI-H2170	Sq	wt	wt	wt	wt	wt	wt	wt	wt	wt
	LK-2	Sq	wt	wt	wt	wt	wt	wt	wt	wt	wt
	SBC-5	SCLC	wt	wt	wt	wt	wt	wt	wt	S1050L^e^	wt
	DMS114	SCLC	wt	wt	wt	wt	wt	wt	T870N^d^	wt	wt

wt: wild type

^a^, E1435 resides downstream of the tyrosine kinase domain (1116–1383) of ALK; its significance is unknown.

^b^, Q61H is a minor mutation in exon 3 of KRAS.

^c^, R77L of RET is located at the N-terminus; its significance is unknown.

^d^, T870N of ROS1 resides outside any known motifs; its significance is unknown.

^e^, S1050L of ERBB2 resides near the Tyr1054 residue downstream of the tyrosine kinase domain; its significance is unknown.

### MAPK signal regulates PD-L1 expression in human lung cancer cells

**Fig 2:**
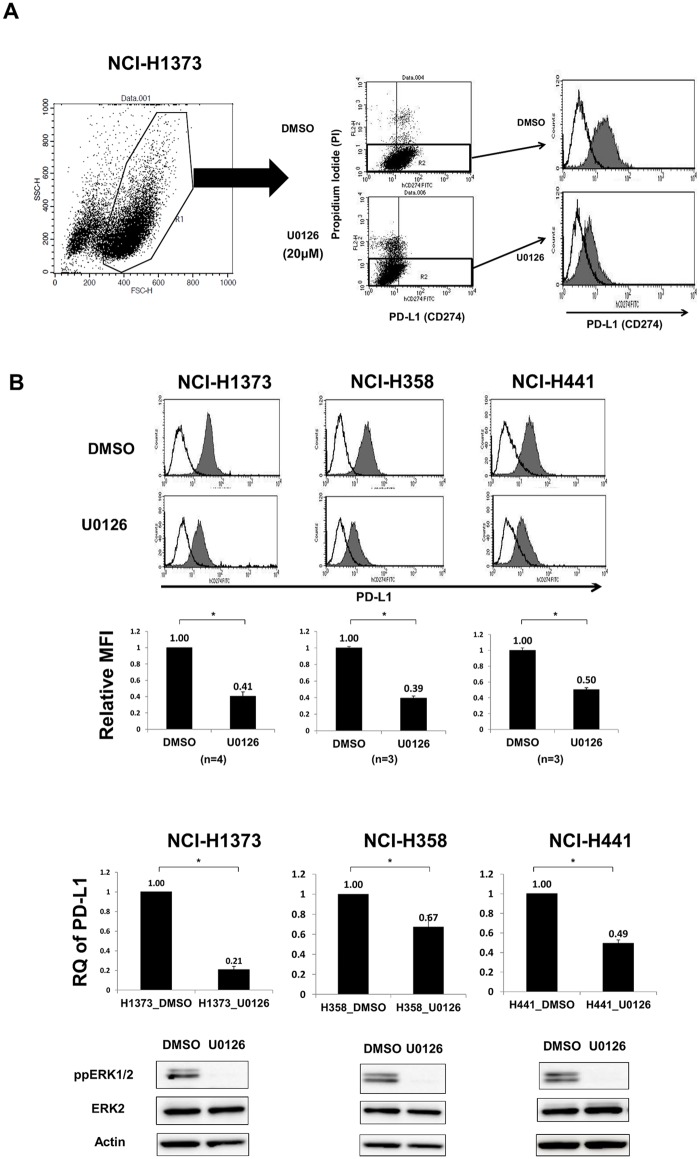
MEK inhibition significantly decreased PD-L1 expression in human lung cancer cell lines. A, Gating strategy for the determination of PD-L1 levels in lung cancer cell lines stained with FITC anti-PD-L1 mAb. For differentiation between live and dead cells, PI exclusion staining was used. B, (upper) Representative histogram of PD-L1 (shaded histogram) of three *KRAS*-mutant lung adenocarcinoma cell lines with DMSO or U0126 (20 μM) for 24 h. Empty histogram indicates the isotype control. (upper middle) Relative MFI (PD-L1 MFI / isotype control MFI) of the three cell lines was significantly decreased with U0126. *, p < 0.0001. (lower middle) qRT-PCR also showed a significant decrease of *PD-L1* mRNA expression with U0126. *, p < 0.05. Data are the mean of three or four independent experiments. (lower) One representative immunoblot of phospho-ERK.

Three *KRAS*-mutant lung adenocarcinoma cell lines (NCI-H1373, NCI-H358, and NCI-H441) were evaluated for PD-L1 expression 24 h after treatment with the MEK inhibitor U0126. Flow cytometry analysis revealed a 50–60% decrease of surface PD-L1 expression with U0126 compared with that with DMSO (Fig 2). Time course experiments showed that PD-L1 suppression with U0126 was similar between 24 and 48 h but weaker at 72 h (data not shown). *PD-L1* mRNA levels also significantly decreased with U0126 (Fig 2B), suggesting that transcriptional regulation under the MAPK signal is important for PD-L1 expression. Immunoblotting indicated a decrease in phospho-ERK levels (Fig 2B) but no change in phospho-AKT (T308) or phospho-STAT3 (Y705) levels (data not shown).

To examine the possible post-transcriptional regulation of PD-L1 under an MAPK signal, *miR-200a*, *miR-200b*, and *miR-200c*, which were previously described as being responsible for the post-transcriptional suppression of PD-L1 in human lung cancers [12], were quantified in the three *KRAS*-mutant lung adenocarcinoma cell lines after DMSO or U0126 treatment (S2 Fig). U0126 treatment significantly increased *miR-200a* expression in one (NCI-H358), *miR-200b* expression in two (NCI-H358 and NCI-H441), and *miR-200c* expression in two (NCI-H1373 and NCI-H358) cell lines, suggesting that the MAPK signal suppresses some miRNAs, counteracting the post-transcriptional suppression of PD-L1 by miRNAs in some cell lines. However, the increase in miRNA expression with U0126 was generally weak (9–37%) and inconsistent among the cell lines. So we cannot conclude that miRNA is universally regulated by MAPK signaling.

U0126 significantly decreased PD-L1 expression at both protein and mRNA levels in the NCI-H1975 lung adenocarcinoma cell line with an *EGFR* mutation, in the EBC-1 lung squamous cell carcinoma cell line without an *EGFR/KRAS/ALK* mutation, and in the NCI-H460 lung large cell carcinoma with a *KRAS* (Q61H) mutation (S3 Fig), suggesting that the MAPK signal contributes to PD-L1 expression irrespective of any histological differences.

**Fig 3:**
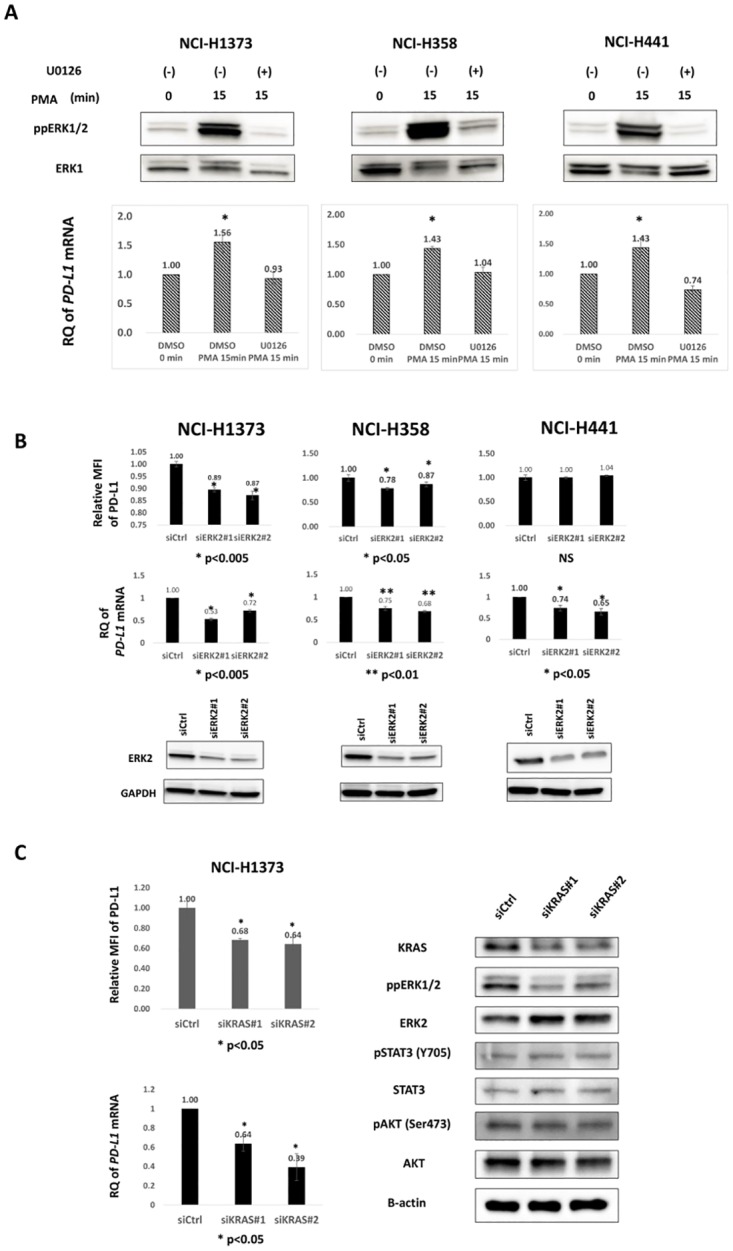
Regulation of PD-L1 by MAPK signaling is supported by PMA stimulation, *ERK2* RNAi, and *KRAS* RNAi. A, Three *KRAS*-mutant lung adenocarcinoma cell lines were serum-starved for 48 h, pre-conditioned with DMSO or U0126 (20μM) for 1hr, then stimulated with 100 nM PMA for 15 min, and then evaluated for phospho-ERK and *PD-L1* mRNA levels. Immunoblot (upper) and qRT-PCR (lower) showed increases in phospho-ERK and *PD-L1* mRNA levels 15 min after PMA stimulation, respectively. *, p < 0.05. Data are the mean of three independent experiments. B, Three *KRAS*-mutant lung adenocarcinoma cell lines were transfected with two siRNAs (#1 and #2) specific for *ERK2* or a control siRNA (siCtrl) at 100 nM. Forty-eight hours after transfection, cells were harvested for analysis. (upper) Flow cytometric analysis showed the significant suppression of surface PD-L1 levels, except in NCI-H441. (middle) qRT-PCR showed a significant decrease in *PD-L1* mRNA levels in all three cell lines. (lower) Immunoblotting indicated a significant decrease in ERK2 protein levels with the two siRNAs. Data are the mean of three independent experiments. C, PD-L1 expression in NCI-H1373 cells was significantly suppressed with two siRNAs specific for *KRAS* (#1 and 2) at both surface protein (upper left) and mRNA levels (lower left). Immunoblotting showed that the KRAS knockdown was associated with the inhibition of ERK phosphorylation (right).

To support the specificity of the MAPK signal for PD-L1 regulation, we subjected the three *KRAS*-mutant lung adenocarcinoma cell lines to PMA stimulation in order to directly activate ERKs, which was associated with a significant increase in *PD-L1* mRNA levels (Fig 3A). Pretreatment with MEK inhibitor U0126 completely abrogated the *PD-L1* mRNA increase, suggesting the PMA dominantly activate MAPK signal. Furthermore, we subjected these cell lines to *ERK2* RNAi and found a significant decrease in PD-L1 expression at both protein and mRNA levels, except for the protein expression of NCI-H441 (Fig 3B). Surface protein suppression was weaker in the *ERK2* RNAi (11–22%) than in the U0126 experiments (50–60%) (Fig 2), which may be explained by the incomplete knockdown of ERK2 (Fig 3B), residual ERK1 activity, and potentially the presence of signal cross-talk between other MAPKKs inhibited by U0126 and cJUN, a transcription factor primarily driven by MAPK signaling. Collectively, these results suggest that MAPK signaling contributes to ectopic PD-L1 expression in *KRAS*-mutant lung cancer cell lines. The contribution of KRAS to downstream MAPK signaling and PD-L1 expression was confirmed by *KRAS* RNAi experiments, in which two siRNAs specific for *KRAS* mRNA led to the significant suppression of PD-L1 as well as ERK phosphorylation, but not of the phosphorylation of AKT or STAT3 (Fig 3C and S4 Fig).

### AP-1 activity downstream of MAPK signal controls PD-L1 expression

**Fig 4:**
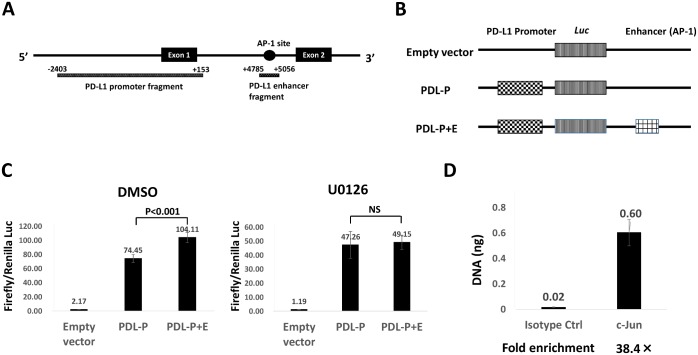
Promoter assay experiments of *PD-L1*. A, Diagrammatic representation of the *PD-L1* regulatory elements, including PD-L1 promoter fragment and the predicted enhancer containing an AP-1 binding site. Fragments cloned into luciferase constructs are annotated below, with positions relative to the *PD-L1* TSS. B, Diagram of luciferase vectors. The empty pGL4.17 vector, the GL4.17 vector with the promoter cloned upstream of the luciferase gene alone (PDL-P), the promoter with the enhancer cloned downstream of the luciferase gene (PDL-P+E). C, *PD-L1* promoter- and enhancer-driven luciferase activity in NCI-H1373 with or without MEK inhibition. Firefly luciferase activity normalized by *Renilla* luciferase activity significantly increased with PDL-P+E compared with that with PDL-P after DMSO treatment (left), while no significant difference was found between PDL-P and PDL-P+E after U0126 treatment (right). Data are representative of three independent experiments with the same results and shown as the mean of quadruplicate experiments. Error bars indicate SD. E, Binding of cJUN to the candidate *PD-L1* enhancer region following ChIP-coupled qPCR. Data are representative of three independent experiments with similar results.

Next, we examined the activity of PD-L1 promoter/enhancer regions. *PD-L1* promoter fragment (−2403 to +153 from TSS) contains the binding sites for transcription factors except AP-1. A candidate AP-1 binding site was found at chr9:5455433–5455446 according to GRCh37/hg19 in the first intron of the *PD-L1* gene by ChIP-seq data from UCSC Genome Browser (Fig 4A). The promoter fragment and a candidate enhancer fragment containing the AP-1 site (+4785 to +5056 from TSS) were cloned to a pGL4.17 luciferase vector, and the *PD-L1* promoter alone (PDL-P) and the *PD-L1* promoter plus a candidate AP-1 enhancer (PDL-P+E) vectors were constructed (Fig 4B). Luciferase assays were conducted in the NCI-H1373 cell line with or without U0126. Luciferase activity was significantly higher in PDL-P+E than in PDL-P (p < 0.001), but this difference was not found with U0126 (Fig 4C), suggesting that MAPK signaling regulates enhancer activity in the candidate AP-1 binding site. We performed a ChIP assay to confirm the binding of an AP-1 component, cJUN, to this region and found significant binding (Fig 4D), demonstrating the regulation of *PD-L1* transcription under MAPK signaling.

### STAT3, but not PI3K, is partially involved in the control of PD-L1 expression

**Fig 5:**
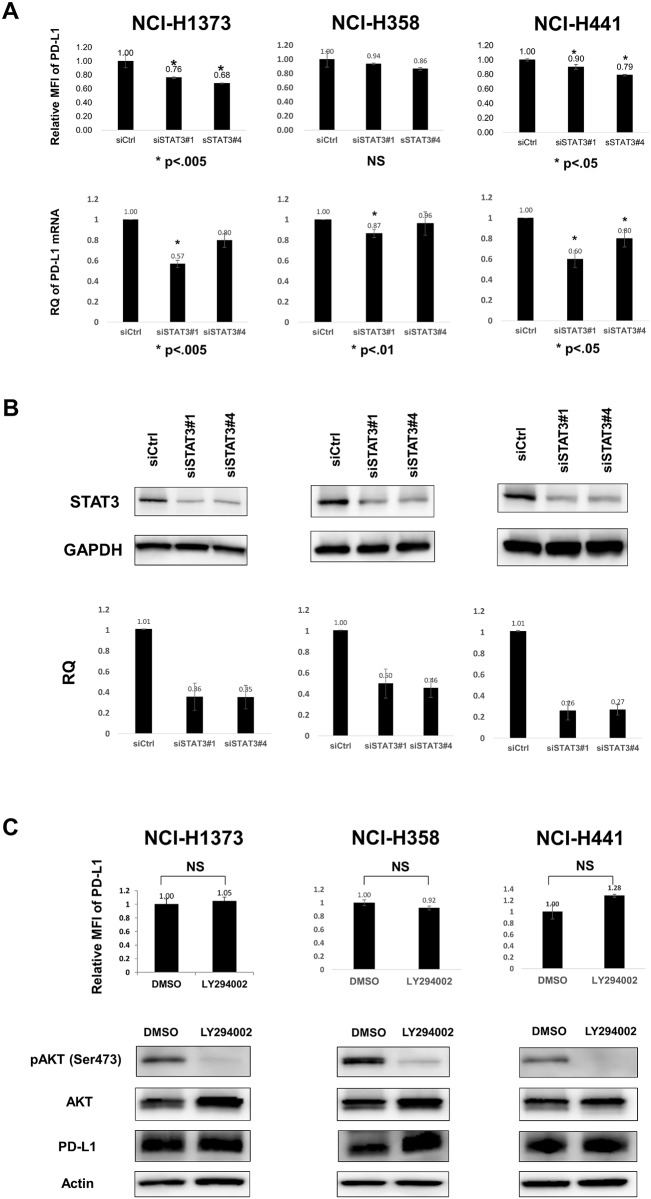
STAT3 signal, but not PI3K signal, partially contributes to ectopic PD-L1 expression in human lung cancer cell lines. A, *STAT3* RNAi reduced PD-L1 expression at both protein (upper) and mRNA (lower) levels in NCI-H1373 and NCI-H441 cells but not in NCI-H358 cells. The experimental conditions were similar to those in Fig 3B. Data are the mean of three independent experiments. B, Immunoblot (upper) and qRT-PCR (lower) of STAT3 showed the decreases in STAT3 protein and mRNA levels, respectively. One representative immunoblot and the mean of three independent qRT-PCR experiments are shown. C, LY294002, a PI3K inhibitor, did not suppress PD-L1 protein expression. Three *KRAS*-mutant lung adenocarcinoma cell lines were treated with DMSO or LY294002 (40 μM) for 24 h. PD-L1 protein levels did not show any significant changes with LY294002 with regard to both the surface (upper) and total protein (lower) levels. Data are the mean of three independent experiments.

Next, we evaluated the contribution of signaling pathways other than MAPK signaling downstream of *KRAS* to ectopic PD-L1 expression. We subjected the three *KRAS*-mutant lung adenocarcinoma cell lines to *STAT3* RNAi, and PD-L1 expression decreased in two of them, NCI-H1373 and NCI-H441, but not in NCI-H358 cells, at both protein and mRNA levels (Fig 5A and 5B). The degree of PD-L1 suppression with *STAT3* RNAi was relatively weak (10–32%) compared with that with U0126, and PD-L1 expression was not affected in one cell line, indicating that the regulatory mechanism of PD-L1 by STAT3 signaling may not occur as widely as MAPK signaling.

PI3K signaling, another downstream feature under *KRAS*, was also evaluated. LY294002, a PI3K inhibitor, did not decrease PD-L1 expression in any of the three *KRAS*-mutant lung adenocarcinoma cell lines (Fig 5C, upper). A time course experiment with LY294002 did not show any decrease in PD-L1 levels from 24 h to 72 h after the treatment (data not shown). Immunoblot (Fig 5C, lower) also showed similar PD-L1 protein levels, ruling out the possibility of an intracellular decrease in the level of PD-L1 protein. These results indicate the partial contribution of STAT3, but not PI3K signaling, to ectopic PD-L1 expression in *KRAS*-mutant human lung adenocarcinoma cell lines. Although our RNAi experiments did not show significant suppression of STAT3 signaling following *KRAS* RNAi (Fig 3C and S4 Fig), which may be explained by the incomplete *KRAS* knockdown, distinct suppression of ERK phosphorylation was observed, indicating the dominant contribution of MAPK signaling to PD-L1 regulation.

### *PD-L1* expression is associated with other biological processes

**Fig 6:**
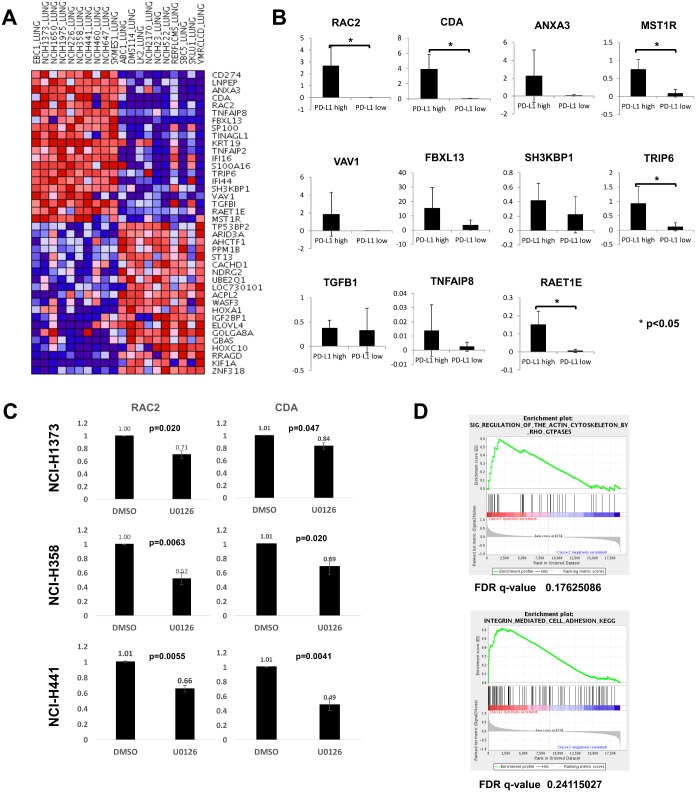
Supervised cluster analysis and GSEA between *PD-L1*-high and -low human lung cancer cell lines suggested mRNA upregulation of several molecules related to cell adhesion, motility, cell proliferation, cell signaling, and pathways related to actin cytoskeleton regulation and integrin-mediated cell adhesion. A, Supervised cluster analysis of 10 *PD-L1*-high versus 10 *PD-L1*-low human lung cancer cell lines. Depicted by the CCLE Analysis Tools: Differential Expression (http://www.broadinstitute.org/ccle/data/browseAnalyses). B, qRT-PCR of 11 selected mRNAs in *PD-L1*-high and -low cell lines. The ΔΔCt method was used for the target mRNA, which was normalized by endogenous *β-actin* mRNA and reference BEAS-2B cells. RQ data are the mean of 10 cell lines. C, *RAC2* and *CDA* mRNA levels were compared between DMSO and U0126 treatments in the three *KRAS*-mutant lung adenocarcinoma cell lines. The target mRNAs were normalized with endogenous *β-actin* mRNAs and DMSO-treated cells as a reference (ΔΔCt method). Data are the mean of three independent experiments. D, GSEA showed significant results for pathways related to actin cytoskeleton regulation by Rho GTPases and to integrin-mediated cell adhesion.

We conducted a supervised cluster analysis between *PD-L1*-high and -low human lung cancer cell lines using CCLE Analysis Tools: Differential Expression. *PD-L1*-high cell lines expressed higher levels of mRNAs related to cell adhesion (*TRIP6* and *SH3KBP1*), motility (*VAV1 and RAC2*), cell proliferation (*ANXA3 and IFI16*), apoptosis (*TNFAIP8* and *SH3KBP1*), cell signaling (*RAC2 and MST1R*,), ubiquitination (*FBXL13*), a ligand for NKG2D (*RAET1E*), or metabolism (*CDA*) than *PD-L1*-low cell lines (Fig 6A). The difference in the expression of some of these genes between *PD-L1*-high and -low human lung cancer cell lines was confirmed by qRT-PCR (Fig 6B and S2 Table). The levels of *RAC2*, *CDA*, *MST1R*, *TRIP6*, and *RAET1E* were significantly higher in *PD-L1*-high than in *PD-L1*-low cell lines. However, because the mRNA levels of *MST1R*, *TRIP6*, and *RAET1E* in *PD-L1*-high cell lines were lower than that in BEAS-2B (RQ = 1), the functional significance of these differences is questionable. Next, we compared the expression of *RAC2* and *CDA* mRNAs between DMSO and U0126 treatments in the three *KRAS*-mutant lung adenocarcinoma cell lines (Fig 6C). Significant suppression of the mRNA levels with U0126 compared with DMSO was found in *RAC2* and *CDA* in all three cell lines. Therefore, *RAC2* and *CDA* as well as *PD-L1* were considered to be regulated by the MAPK signaling pathway. RAC2 is a small GTPase regulating diverse types of signal transduction related to the organization of actin filament and regulation of cell proliferation or lamellipodium assembly [20]. CDA is an enzyme involved in pyrimidine metabolism, catalyzing the irreversible hydrolytic deamination of cytidine and deoxycytidine to uridine and deoxyuridine, respectively [21]. It also inactivates gemcitabine, a chemotherapeutic agent against NSCLCs.

GSEA showed that two pathways, actin cytoskeleton regulation by Rho GTPases and integrin-mediated cell adhesion, significantly correlated to the *PD-L1*-high cell lines (Fig 6D). Both supervised cluster analysis and GSEA indicated the possible relevance of enhanced cell motility to increased *PD-L1* expression, and a common pathway(s) regulating these molecules seems to be, at least in part, MAPK signaling.

## Discussion

The mechanisms of ectopic PD-L1 expression in human cancers have been studied in lung cancers [10–13], melanomas [14,15], malignant lymphomas [16–18], myelomas [19], and gliomas [9]. Oncogenic driver mutations, such as mutated *EGFR* [10,11], *EML4-ALK* [13], or *NPM-ALK* [16], with activated downstream signals, have been shown to contribute to ectopic PD-L1 expression. These observations suggest that some constitutively activated signals are targetable immune evasion signals, as previously indicated [6–8]. It has been reported that various signals including MAPK [13,14,17–19], STAT3 [16], or PI3K [9, 13, 14] are relevant to PD-L1 expression; however, the contribution of each signal is inconsistent among different cancers. In NSCLCs, *EGFR* mutation [10,11] and *EML4-ALK* fusion [13] have been shown to be related to PD-L1 expression. Here we reported that *KRAS* mutation also contributes to enhanced PD-L1 expression. Ota et al. showed that an MEK inhibitor (U0126) and a PI3K inhibitor (LY294002), but not a STAT3 inhibitor (S3I–201), decrease PD-L1 expression in *EGFR*-mutant and *EML4-ALK*-positive lung cancer cells, as determined by qRT-PCR and flow cytometry [13]. However, direct evidence of the transcriptional regulation of PD-L1 by MAPK signaling has not yet been obtained. We demonstrated that PD-L1 enhancer activity is driven by AP-1 under MAPK signaling and the binding of cJUN to the candidate AP-1 site in a human *KRAS*-mutant adenocarcinoma cell line. Contrary to the previous report mentioned above, our analysis showed the partial contribution of STAT3, but not PI3K signaling, to PD-L1 expression. The different mutational status may lead to different signaling pathways for PD-L1 expression.

Our lung cancer panel of *PD-L1*-high cell lines included three squamous cell carcinomas without any known oncogenic driver mutations and one large cell carcinoma with a *KRAS* (Q61H) mutation (Table 1). However, the MEK inhibitor U0126 significantly reduced PD-L1 levels in these cell lines (S3 Fig), suggesting that MAPK signaling is involved in ectopic PD-L1 expression irrespective of any histological differences, even without any known oncogenic driver mutations.

Our data using *KRAS*-mutated lung cancer cell lines may suggest the translational importance of this mechanistic link in clinical lung cancer tissues. A correlation between PD-L1 expression and the status of *KRAS* mutation in larger sets of laser microdissected clinical lung tumor cells should be warranted.

Ota et al. [13] and we could not identify the complete loss of PD-L1 expression with a MEK inhibitor, suggesting that MAPK signaling only contributes to the enhancer activity of *PD-L1*, which was supported by our luciferase assay results showing substantial transcriptional activity in the promoter fragment (-2403 to +153 from TSS) without AP-1 sites (PDL-P, Fig 4C). This promoter sequence includes potential binding sites for other transcription factors, such as IRF-1 [22], NF-κB [23], STAT-3 [24], and HIF-1α [25]. Therefore, PD-L1 expression seems to depend not only on MAPK signaling but also on other regulatory signals, for which further analysis is warranted.

Two *KRAS*-mutant lung adenocarcinoma cell lines, SK-LU-1 and NCI-H23, did not express PD-L1 at both mRNA and protein levels (Fig 1, S1 Table and S1 Fig). Akbay et al. reported that the forced expression of mutant *KRAS* (G12V) in BEAS-2B does not induce PD-L1 expression [10], suggesting that the mutant *KRAS* is insufficient for PD-L1 expression. Skoulidis et al. recently reported three subsets (KL, KP, and KC subtypes) of *KRAS*-mutant lung adenocarcinoma cell lines according to the co-occurring genetic events as determined by an integrative analysis of genomic, transcriptomic, and proteomic data [26]. These subsets showed biologically and therapeutically relevant differences, and KP tumors exhibited higher levels of somatic mutations, immune checkpoint effector molecules including PD-L1, and improved relapse-free survival. The inconsistency in PD-L1 expression among the *KRAS*-mutant lung adenocarcinoma cell lines may reflect their heterogeneity.

We identified *RAC2* and *CDA* as genes with significantly higher expression in *PD-L1*-high lung cancer cell lines. The expression of these genes as well as *PD-L1* was dependent on MAPK signaling, suggesting that MAPK signaling constitutes a common pathway for an immune checkpoint molecule, and other functional molecules regulating cell motility, proliferation, or pyrimidine metabolism. GSEA identified that the pathways of the actin cytoskeleton regulation by Rho GTPases and integrin-mediated cell adhesion are significantly related to *PD-L1*-high lung cancer cell lines. These signatures may be related to the enhanced cellular motility or invasive ability and are reminiscent of the findings in a previous report by Chen et al. describing the correlation between PD-L1 expression and epithelial—mesenchymal transition (EMT) [12]. We also found the substantial expression of several chemokines (CXCL1, CXCL5, CXCL8, and CX3CL1) related to cell migration, invasion, metastasis, or EMT [27–30], in *KRAS*-mutant lung adenocarcinoma cell lines, which was also dependent on MAPK signaling (unpublished data). Although the functional significance of these molecules requires further investigation, we speculate that MAPK signaling regulates gene expression related to EMT and immune evasion. Furthermore, we found CCL17 (TARC) expression was higher, while CXCL3 expression was lower in PD-L1-high cell lines than in PD-L1-low cell lines, respectively (S5 Fig). CCL17 is related to the recruitment of Th2 cells and regulatory T cells, and CXCL3 can recruit neutrophils and fibroblasts. Functional significance of these chemokine profiles needs further evaluation.

In summary, this study showed that PD-L1 expression is regulated by MAPK and partially by STAT3 signaling in *KRAS*-mutant lung adenocarcinoma cell lines. We also demonstrated the MAPK-dependent transcriptional activity of AP-1, as well as the binding of cJUN to AP-1, in the *PD-L1* gene. MAPK signaling also regulates PD-L1 in squamous and large-cell lung carcinoma cell lines. PD-L1 is correlated with the expression of other functional genes (*RAC2* and *CDA*) and to pathways related to the cytoskeleton and cell adhesion, suggesting possible implications for EMT.

## Supporting Information

S1 Fig Flow cytometric analysis of human lung cancer cell lines. A, Ten *PD-L1*-high cell lines (RQ > 2.7 in Fig 1). B, Ten *PD-L1*-low cell lines (RQ < 1). Empty and shaded histograms indicate isotype control and PD-L1, respectively. BEAS-2B, a normal human bronchial cell line, is shown as a reference.
(PDF)

S2 Fig Increase in micro-RNA expression with MEK inhibition. qRT-PCR (ΔΔCt method) of *KRAS*-mutant lung adenocarcinoma cell lines showed inconsistent increases in the expression of *miR-200a*, *miR-200b*, and *miR-200c* with U0126 (20 μM, 24 h). Each miRNA level was normalized by *miR-16* (an internal control) and DMSO-treated cells (reference). Data are the mean of three independent experiments.
(PDF)

S3 Fig MEK inhibition significantly decreased the PD-L1 expression of *EGFR*-mutant lung adeno-, squamous cell, or large cell carcinomas at both protein and mRNA levels. (A) NCI-H1975, an adenocarcinoma cell line harboring *EGFR* mutation (L858R, T790M); (B) EBC-1, a squamous cell carcinoma cell line; and (C) NCI-H460, a large cell carcinoma cell line. Data are the mean of three independent experiments.
(PDF)

S4 Fig *KRAS* RNAi decreased PD-L1 expression in two *KRAS*-mutant lung adenocarcinoma cell lines, NCI-H358 and NCI-H441. A, Surface protein (relative MFI) (upper) and mRNA levels (RQ) (lower) of PD-L1. Data are the mean of three independent experiments. B, Immunoblot shows KRAS knockdown and decreased ERK phosphorylation. One representative result of two or three independent experiments.
(PDF)

S5 Fig Supervised cluster analysis of chemokine expression between PD-L1-high and -low human lung cancer cell lines. CCL17 expression is higher, while CXCL3 expression is lower in PD-L1-high than in PD-L1-low lung cancer cell lines.
(PDF)

S1 Table PD-L1 RQ and MFI of human lung cancer cell lines. (XLSX)

S2 Table Primer sets for qRT-PCR. (XLSX)
