# Supplementary figures and images for: RAS–Mitogen-Activated Protein Kinase Signal Is Required for Enhanced PD-L1 Expression in Human Lung Cancers
Source: PLoS One. 2016 Nov 15;11(11):e0166626. doi: 10.1371/journal.pone.0166626 (PMC5112979; doi:10.1371/journal.pone.0166626)

**A**

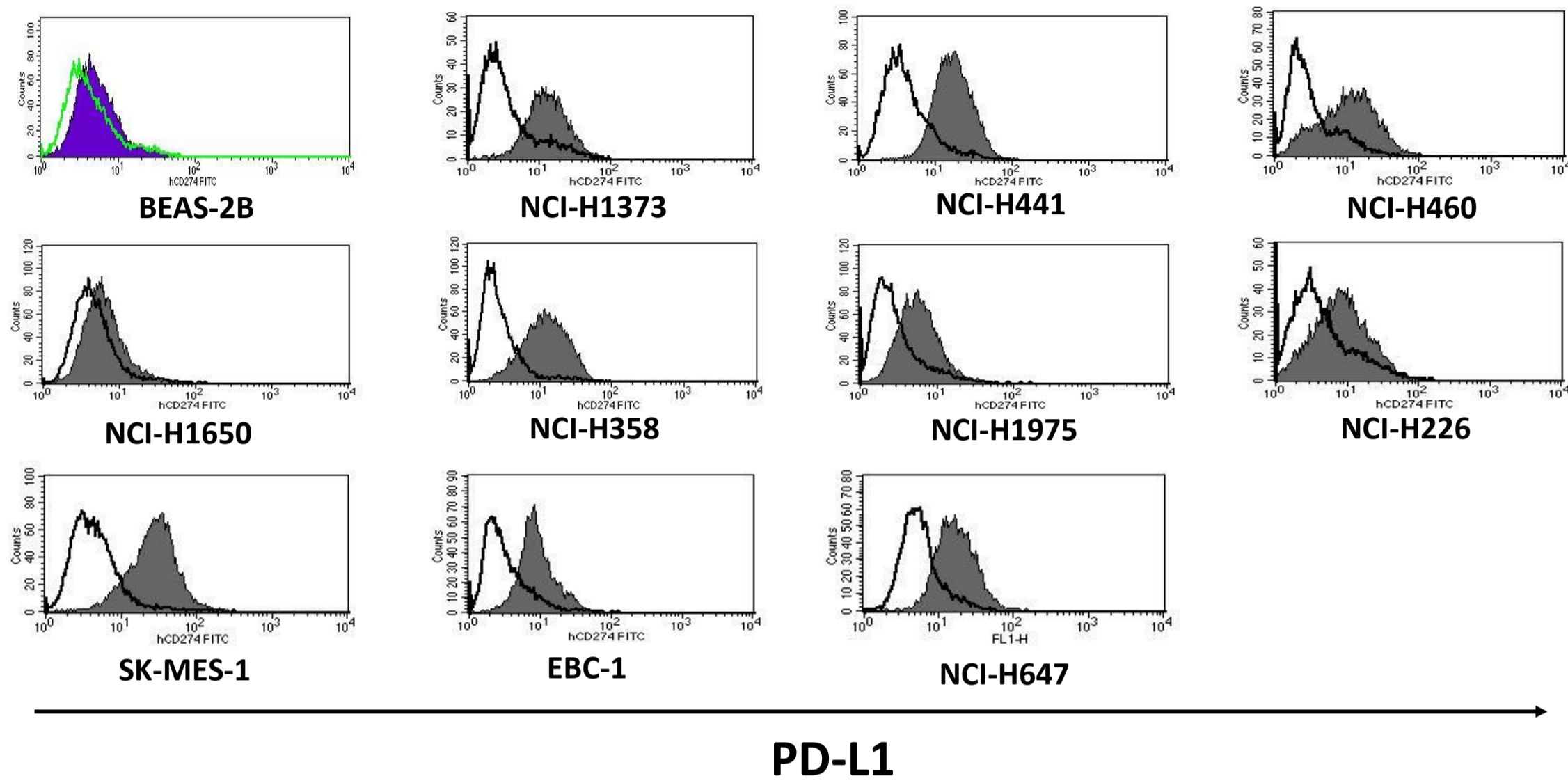

**B**

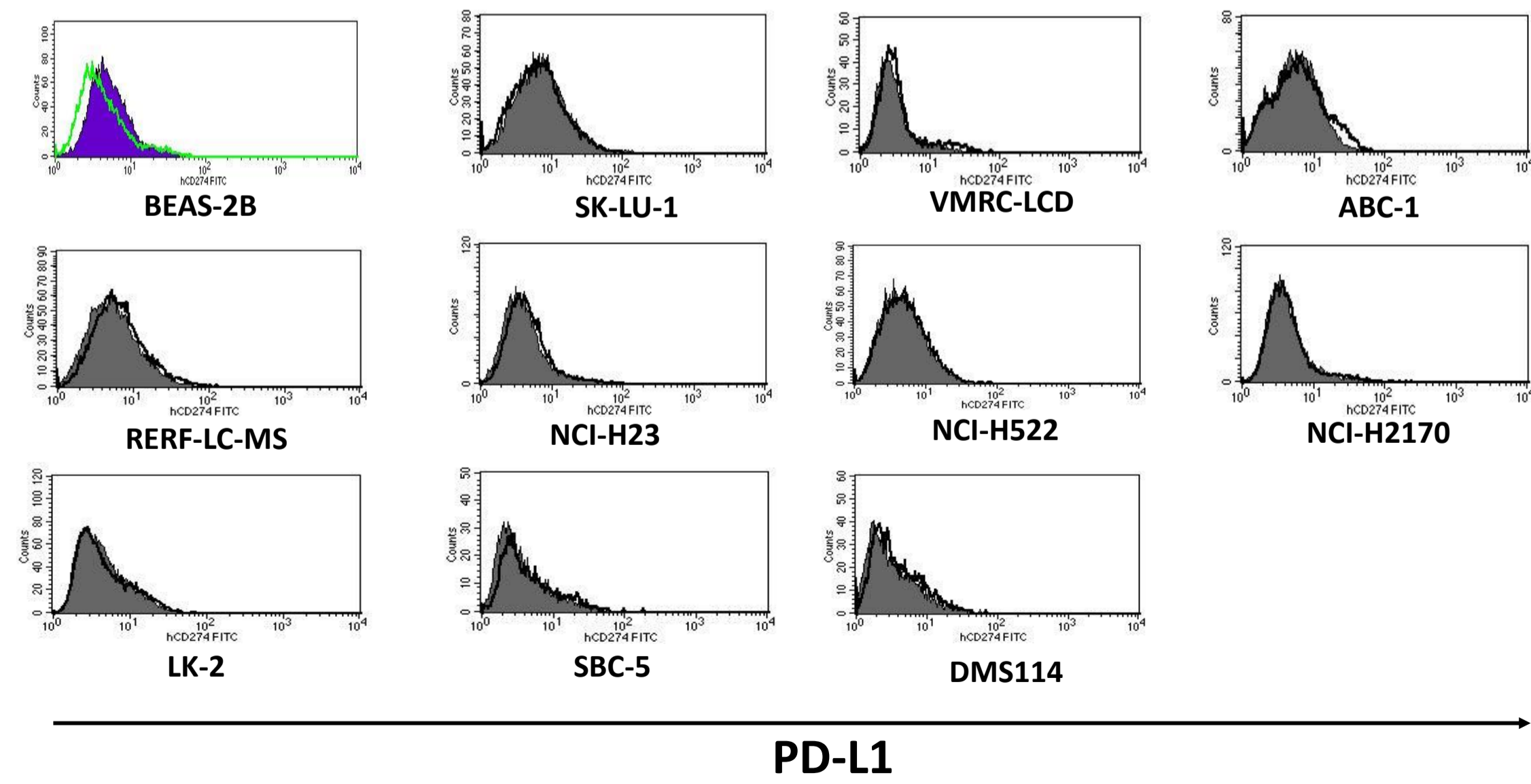

Supplement: S1 Fig — A, Ten PD-L1-high cell lines (RQ > 2.7 in Fig 1). B, Ten PD-L1-low cell lines (RQ < 1). Empty and shaded histograms indicate isotype control and PD-L1, respectively. BEAS-2B, a normal human bronchial cell line, is shown as a reference. (PDF) [file pone.0166626.s001.pdf]

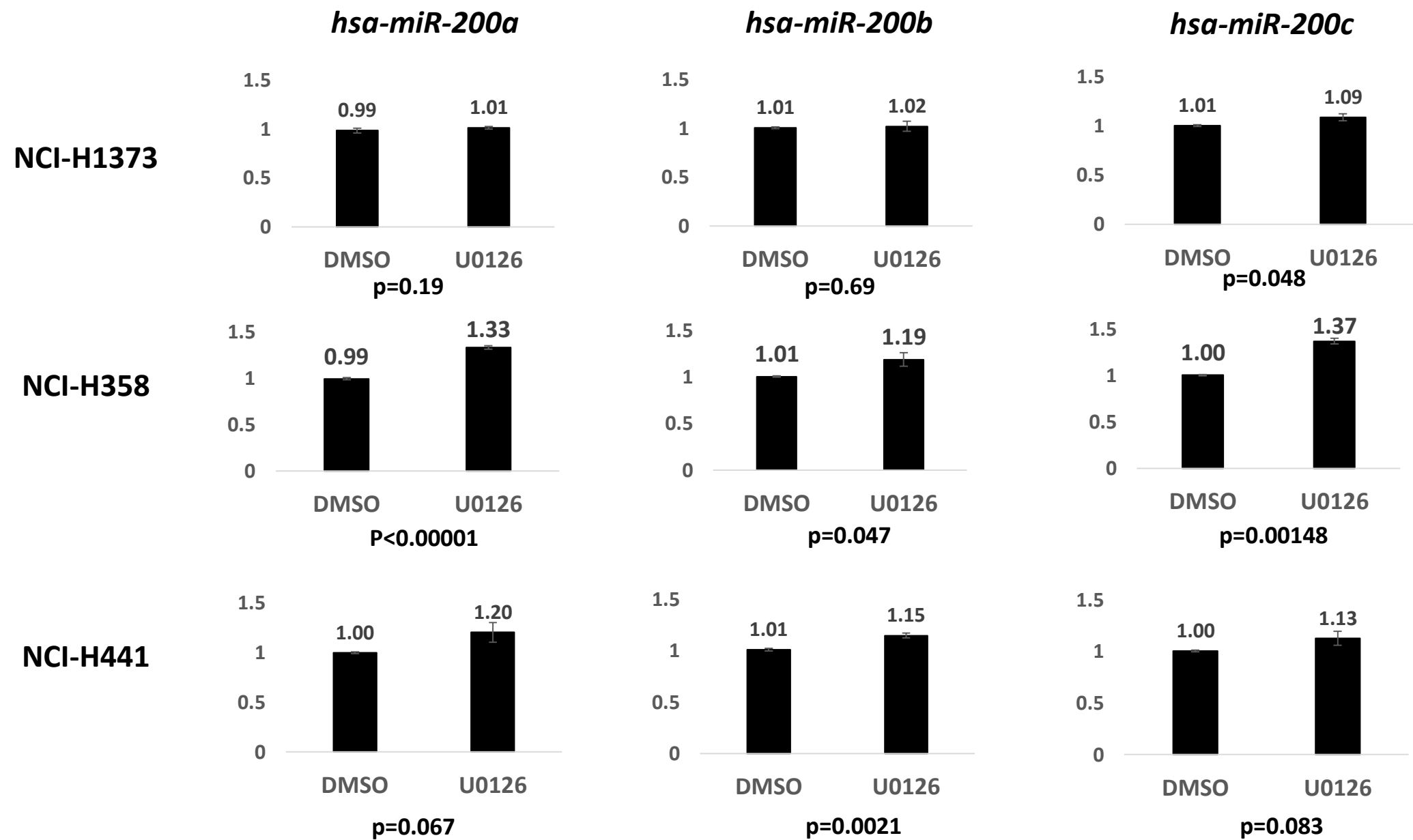

S2 Fig. Sumimoto, et al.

Supplement: S2 Fig — qRT-PCR (ΔΔCt method) of KRAS-mutant lung adenocarcinoma cell lines showed inconsistent increases in the expression of miR-200a, miR-200b, and miR-200c with U0126 (20 μM, 24 h). Each miRNA level was normalized by miR-16 (an internal control) and DMSO-treated cells (reference). Data are the mean of three independent experiments. (PDF) [file pone.0166626.s002.pdf]

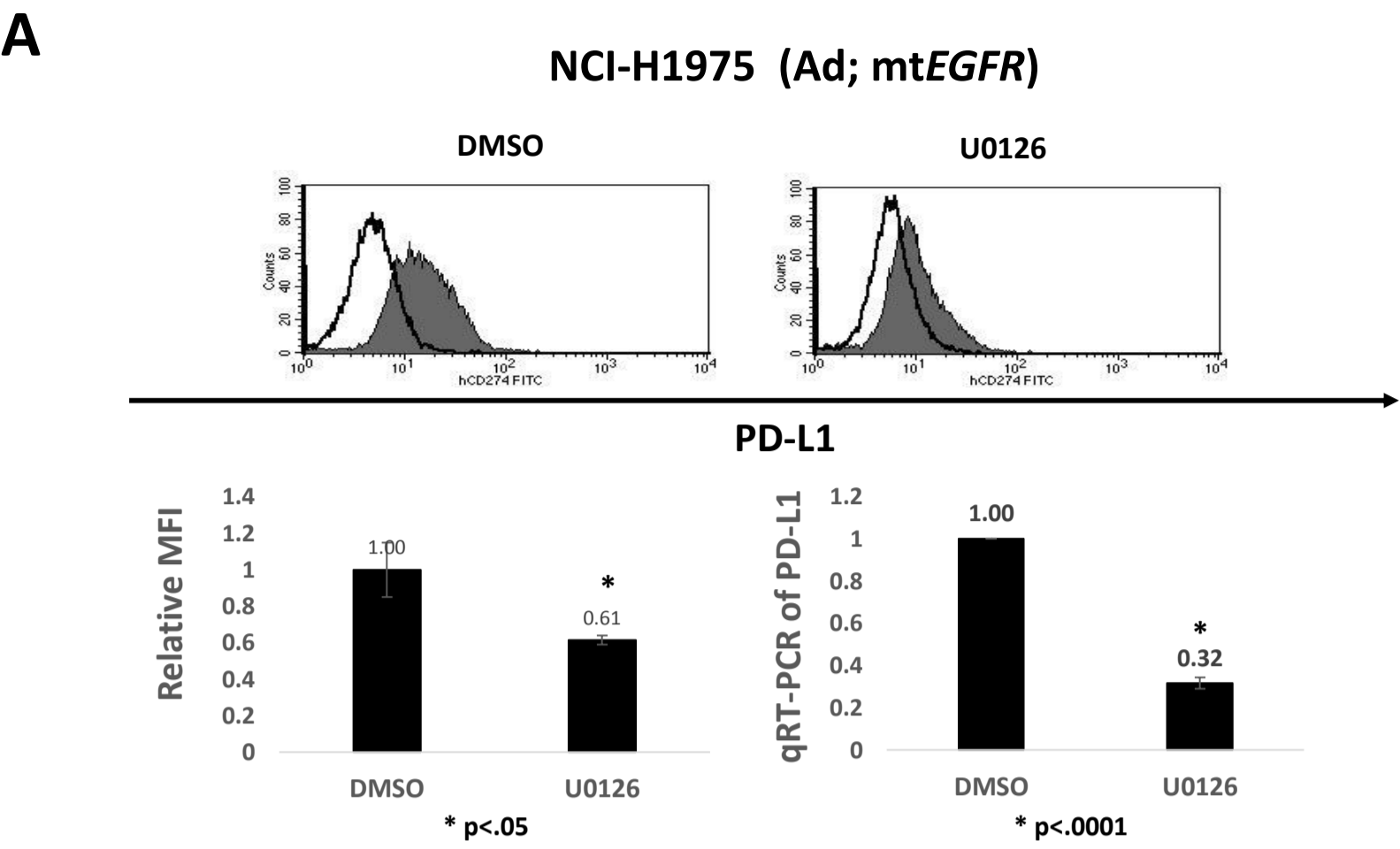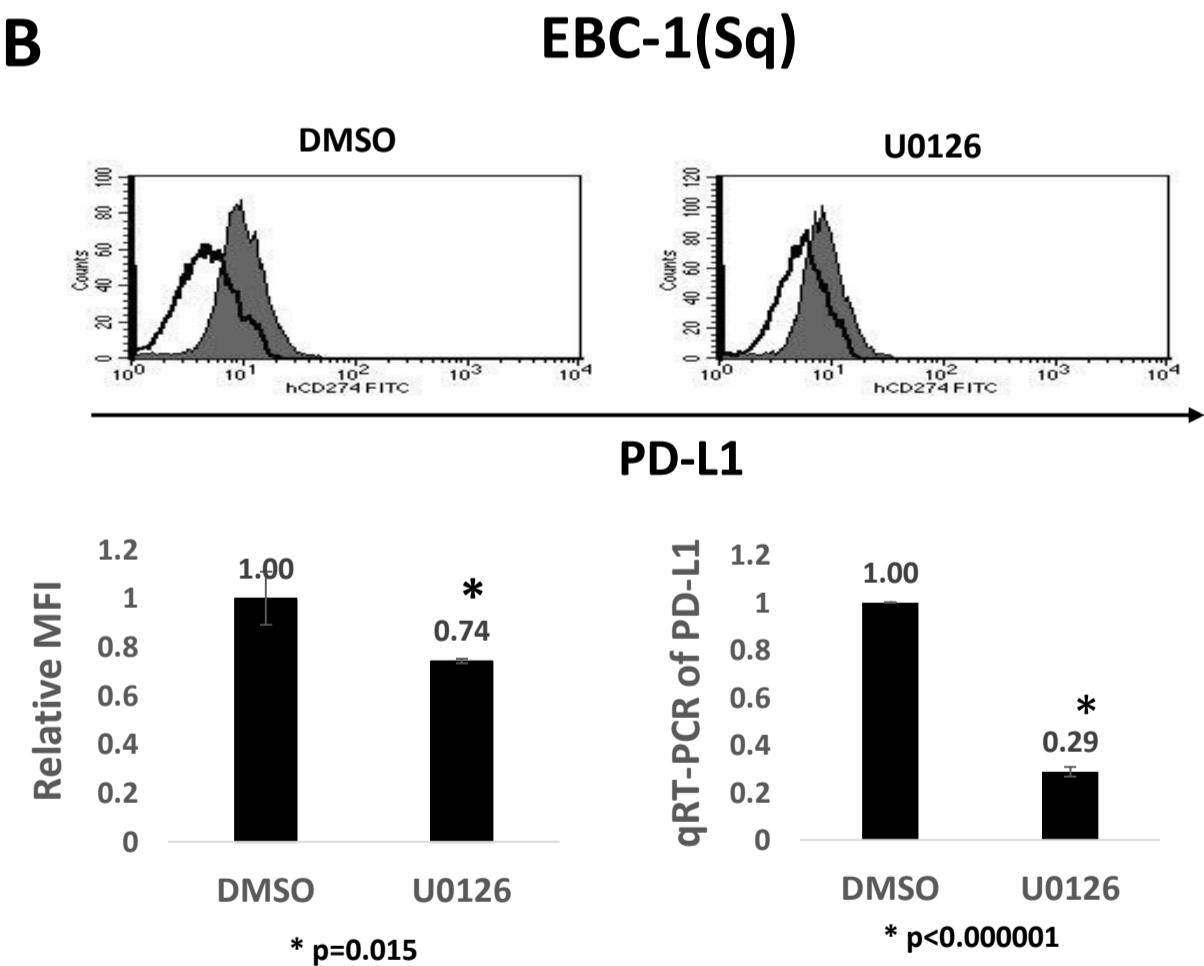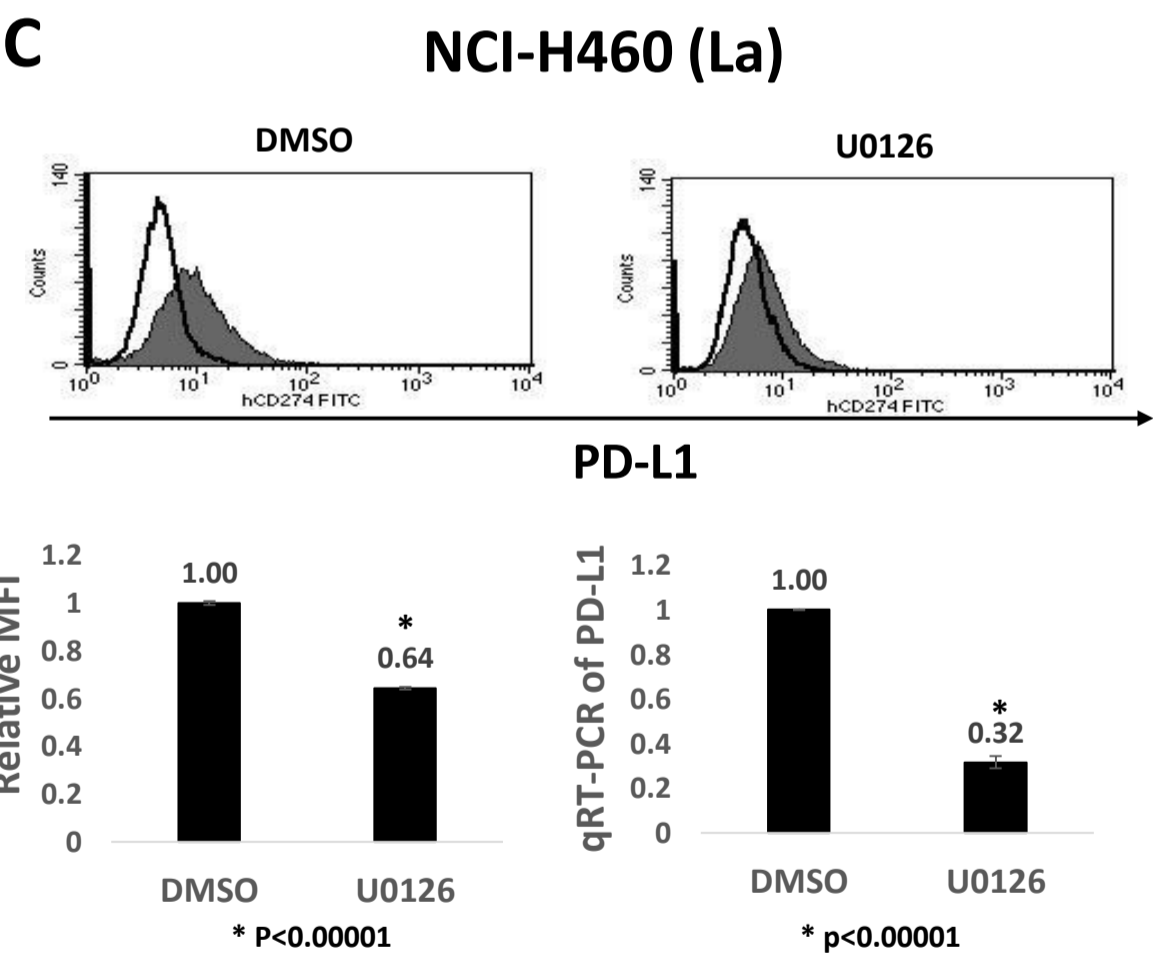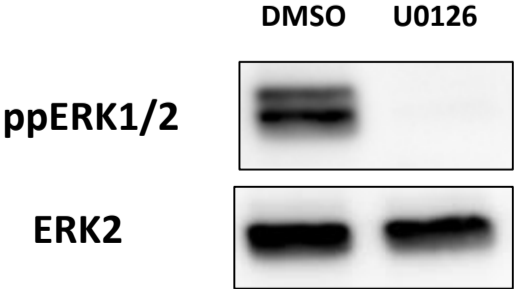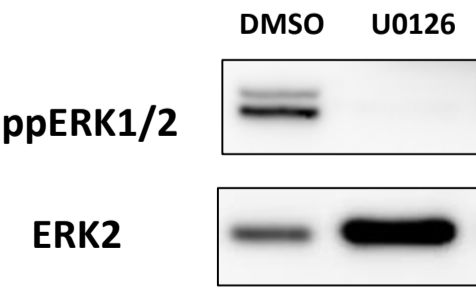

S3 Fig. Sumimoto, et al.

Supplement: S3 Fig — (A) NCI-H1975, an adenocarcinoma cell line harboring EGFR mutation (L858R, T790M); (B) EBC-1, a squamous cell carcinoma cell line; and (C) NCI-H460, a large cell carcinoma cell line. Data are the mean of three independent experiments. (PDF) [file pone.0166626.s003.pdf]

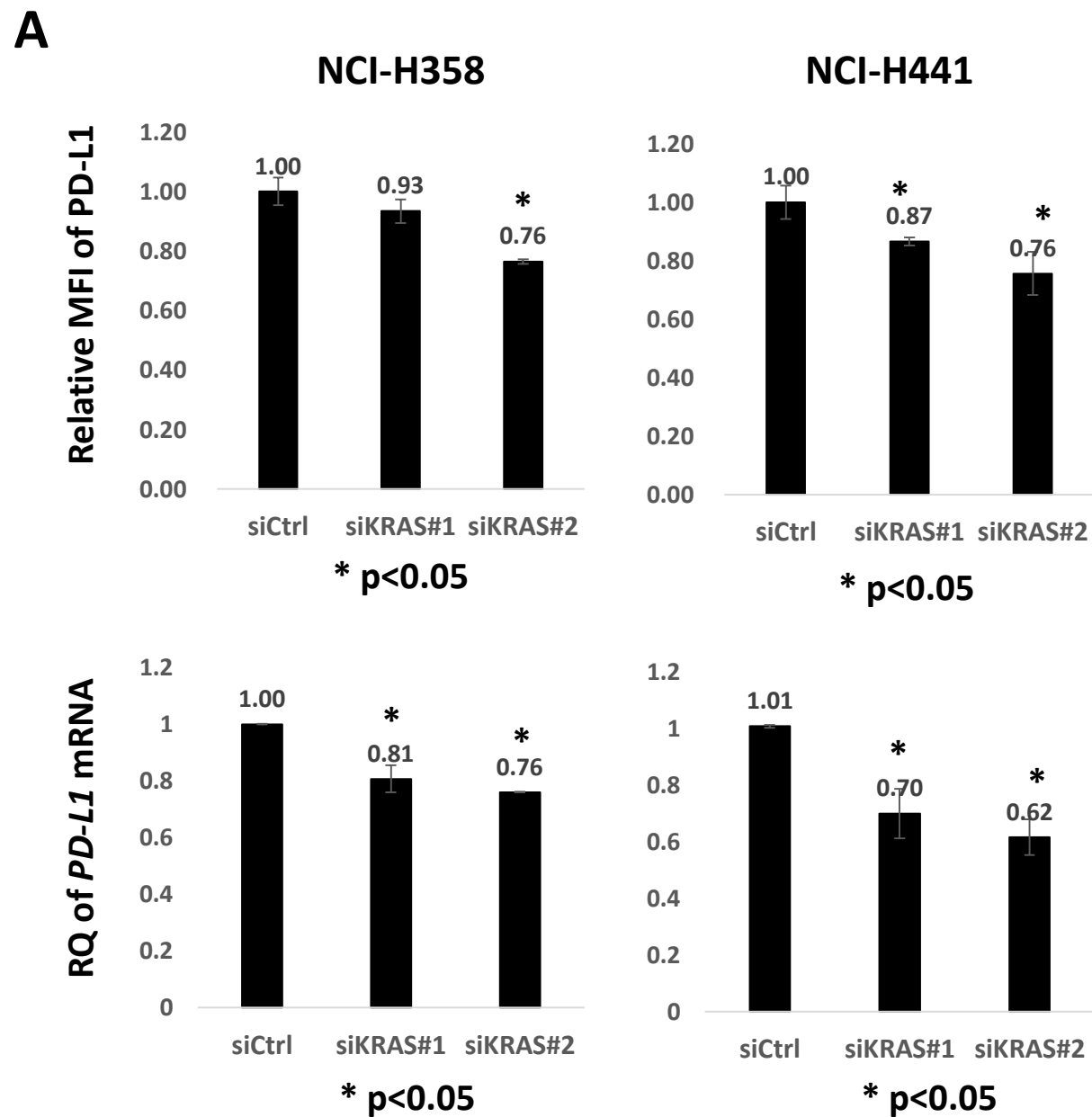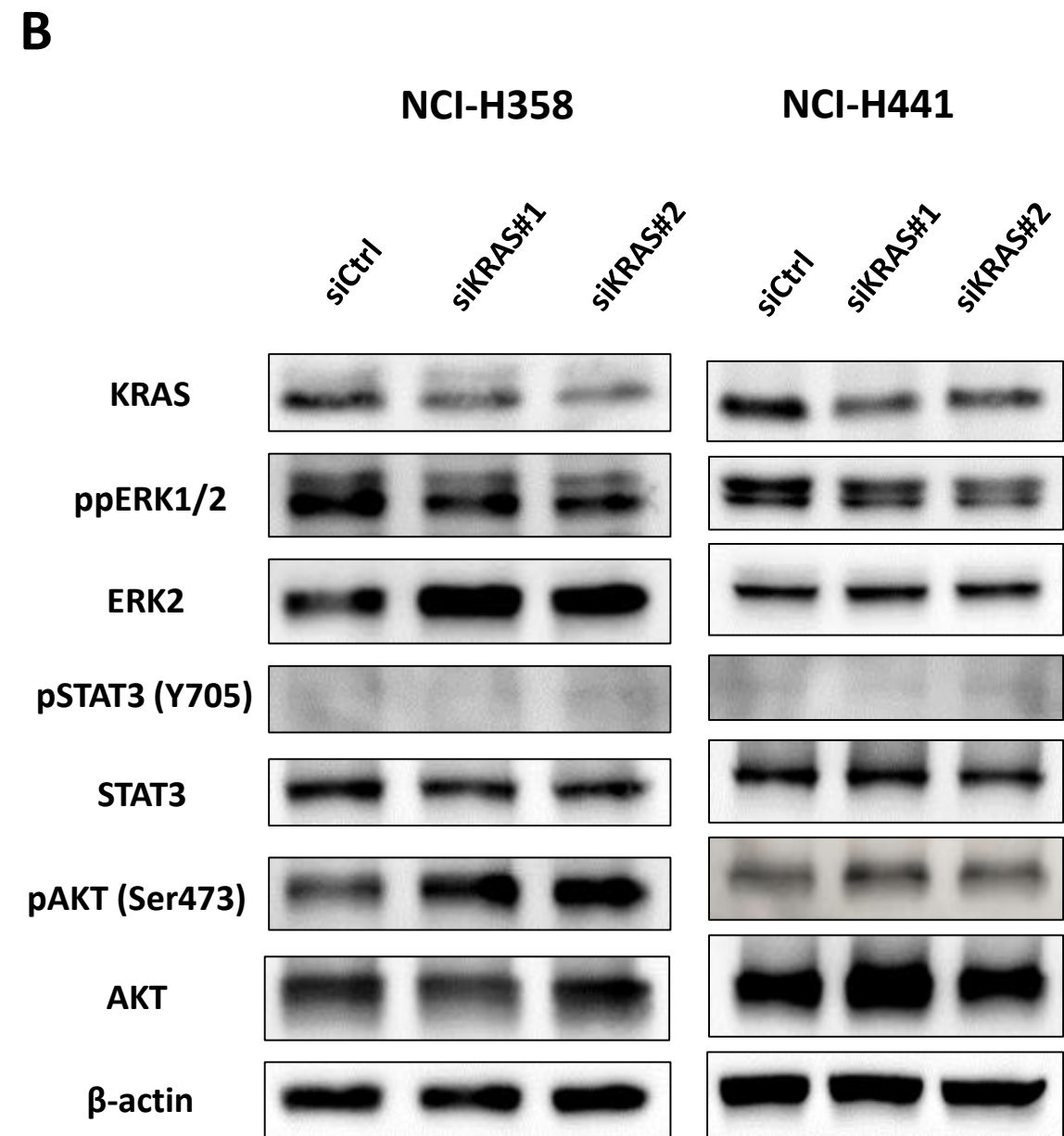

Supplement: S4 Fig — A, Surface protein (relative MFI) (upper) and mRNA levels (RQ) (lower) of PD-L1. Data are the mean of three independent experiments. B, Immunoblot shows KRAS knockdown and decreased ERK phosphorylation. One representative result of two or three independent experiments. (PDF) [file pone.0166626.s004.pdf]
