# Supplementary material for: RAS–Mitogen-Activated Protein Kinase Signal Is Required for Enhanced PD-L1 Expression in Human Lung Cancers
Source: PLoS One. 2016 Nov 15;11(11):e0166626. doi: 10.1371/journal.pone.0166626 (PMC5112979; doi:10.1371/journal.pone.0166626)
Supplement: S5 Fig — CCL17 expression is higher, while CXCL3 expression is lower in PD-L1-high than in PD-L1-low lung cancer cell lines. (PDF) [file pone.0166626.s005.pdf]

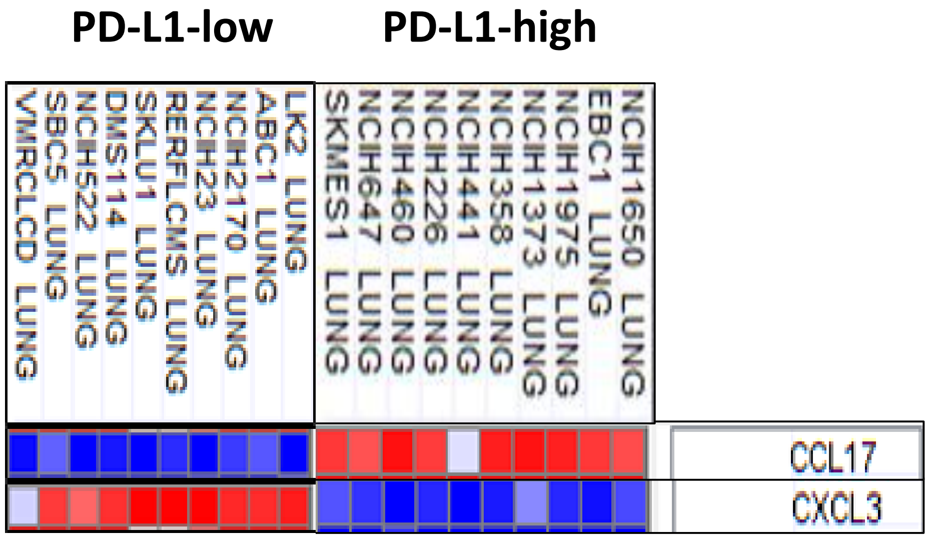
PD-L1-low
PD-L1-high
VMRCLCD LUNG
SBC5 LUNG
NCIH522 LUNG
DMS114 LUNG
SKLU1 LUNG
RERFLCMS LUNG
NCIH23 LUNG
NCIH2170 LUNG
ABC1 LUNG
LK2 LUNG
SKMES1 LUNG
NCIH647 LUNG
NCIH460 LUNG
NCIH226 LUNG
NCIH441 LUNG
NCIH358 LUNG
NCIH1373 LUNG
NCIH1975 LUNG
EBC1 LUNG
NCIH1650 LUNG
CCL17
CXCL3
